# Arterial spin labeling perfusion cardiovascular magnetic resonance of the calf in peripheral arterial disease: cuff occlusion hyperemia vs exercise

**DOI:** 10.1186/s12968-015-0128-y

**Published:** 2015-02-22

**Authors:** David Lopez, Amy W Pollak, Craig H Meyer, Frederick H Epstein, Li Zhao, Arthur J Pesch, Ronny Jiji, Jennifer R Kay, Joseph M DiMaria, John M Christopher, Christopher M Kramer

**Affiliations:** Department of Medicine, University of Virginia Health System, Charlottesville, VA USA; Department of Radiology and Medical Imaging, University of Virginia Health System, Charlottesville, VA USA; Department of Biomedical Engineering, University of Virginia Health System, Charlottesville, VA USA; Cardiovascular Imaging Center, University of Virginia Health System, Charlottesville, VA USA

**Keywords:** Peripheral arterial disease, Cardiovascular magnetic resonance, Perfusion, Arterial spin labeling, Cuff occlusion hyperemia

## Abstract

**Background:**

Assessment of calf muscle perfusion requires a physiological challenge. Exercise and cuff-occlusion hyperemia are commonly used methods, but it has been unclear if one is superior to the other. We hypothesized that post-occlusion calf muscle perfusion (Cuff) with pulsed arterial spin labeling (PASL) cardiovascular magnetic resonance (CMR) at 3 Tesla (T) would yield greater perfusion and improved reproducibility compared to exercise hyperemia in studies of peripheral arterial disease (PAD).

**Methods:**

Exercise and Cuff cohorts were independently recruited. PAD patients had an ankle brachial index (ABI) between 0.4-0.9. Controls (NL) had no risk factors and ABI 0.9-1.4. Subjects exercised until exhaustion (15 NL-Ex, 15 PAD-Ex) or had a thigh cuff inflated for 5 minutes (12 NL-Cuff, 11 PAD-Cuff). Peak exercise and average cuff (Cuff_*mean*_) perfusion were compared. Six participants underwent both cuff and exercise testing. Reproducibility was tested in 8 Cuff subjects (5 NL, 3 PAD).

**Results:**

Controls had greater perfusion than PAD independent of stressor (NL-Ex 74 ± 21 vs. PAD-Ex 43 ± 10, p = 0.01; NL-Cuff_*mean*_ 109 ± 39 vs. PAD-Cuff_*mean*_ 34 ± 17 ml/min-100 g, p < 0.001). However, there was no difference between exercise and Cuff_*mean*_ perfusion within groups (p > 0.6). Results were similar when the same subjects had the 2 stressors performed. Cuff_*mean*_ had superior reproducibility (Cuff_*mean*_ ICC 0.98 vs. Exercise ICC 0.87) and area under the receiver operating characteristic curve (Cuff_*mean*_ 0.992 vs. Exercise 0.905).

**Conclusions:**

Cuff hyperemia differentiates PAD patients from controls, as does exercise stress. Cuff_*mean*_ and exercise calf perfusion values are similar. Cuff occlusion hyperemia has superior reproducibility and thus may be the preferred stressor.

## Background

Peripheral arterial disease (PAD) affects more than 8 million adults in the U.S. [1]. Those affected with PAD have impaired exercise tolerance, progressive functional decline, reduced quality of life [2] and increased cardiovascular mortality [3]. PAD extends beyond large vessel stenosis to impaired skeletal muscle perfusion [4]. The ankle-brachial index (ABI) is a clinical non-invasive measure of PAD with excellent diagnostic and prognostic value [5]. However, abnormal blood flow to the lower extremity measured by an ABI < 0.90 does not correlate well with time to initial claudication or maximum claudication distance [6].

Our group has shown that contrast-enhanced perfusion cardiovascular magnetic resonance (CMR) of the calf muscle at peak exercise using a plantar-flexion ergometer reproducibly discriminates PAD patients from healthy volunteers independent of exercise workload [7] and that it correlates with walking distance [8]. One important limitation of contrast enhanced CMR using gadolinium chelates is the risk of nephrogenic systemic fibrosis in patients with advanced chronic kidney disease (CKD)(Glomerular filtration rate < 30 ml/min/1.73 m^2^) [9], which commonly coexists with PAD [10].

Arterial spin labeling (ASL) is a CMR technique capable of quantifying perfusion in a spatially and temporally resolved fashion on a ml/min-100 g basis without the use of exogenous contrast [11,12]. Its application to calf perfusion has been validated against venous occlusion plethysmography [13]. Wu et al. used a continuous ASL technique in combination with cuff occlusion to measure post-ischemic reactive hyperemia in the calf using the endpoints of peak hyperemic perfusion and time to peak perfusion [14]. Our group recently showed that peak exercise pulsed ASL differentiates PAD patients with claudication from healthy (NL) subjects with excellent test-retest and inter-observer reproducibility [15]. Similar to our findings with CMR perfusion, the difference remained even when healthy subjects matched PAD patients’ exercise time.

Cuff studies would facilitate quantitative perfusion measurements in those unable to exercise due to critical limb ischemia (CLI), severe claudication, or arthritis. Additionally, cuff studies allow the assessment of hyperemic response in muscles not typically used during ankle flexion exercise. Compared to exercise, cuff occlusion hyperemia is an effort-independent stress, which may further improve reproducibility and maximize the hyperemic response. We hypothesized that Cuff would yield higher perfusion values and improved reproducibility in PAD and healthy subjects compared to Exercise.

## Methods

### Study design

The Exercise cohort (15 NL; 15 PAD) was enrolled between 9/2009 – 7/2011 using similar criteria and their results were previously published [15], although a new analysis method was applied to the data in the present study. The Cuff cohort (12 NL; 11 PAD) was independently recruited between 9/2012 – 5/2014. Healthy subjects without known cardiovascular disease between the ages of 50 and 85 were enrolled. They had no history of tobacco use, hyperlipidemia or diabetes, were free from any exercise-induced symptoms and had an ABI between 0.9 – 1.4. PAD patients were recruited from the University of Virginia (UVA) vascular lab and were between the ages of 30 and 85 years with symptoms of intermittent claudication and ABI between 0.4 – 0.9 measured during the screening period. Exclusion criteria included rest pain, CLI, inability to perform ankle flexion exercise, contraindication to CMR, pregnancy, recent lower extremity percutaneous intervention (<3 months).

Six participants (4 NL, 2 PAD) underwent both cuff occlusion and exercise testing for within subject comparisons of the methods. Exercise test-retest reproducibility was previously reported [15]. To test reproducibility of cuff occlusion, repeat studies on different days were performed in 8 subjects (5 NL, 3 PAD).

All subjects provided written informed consent prior to study enrollment. The study protocol was approved by the Human Investigation Committee of the UVA Health System.

### Study protocol

#### CMR protocol

**Figure 1:**
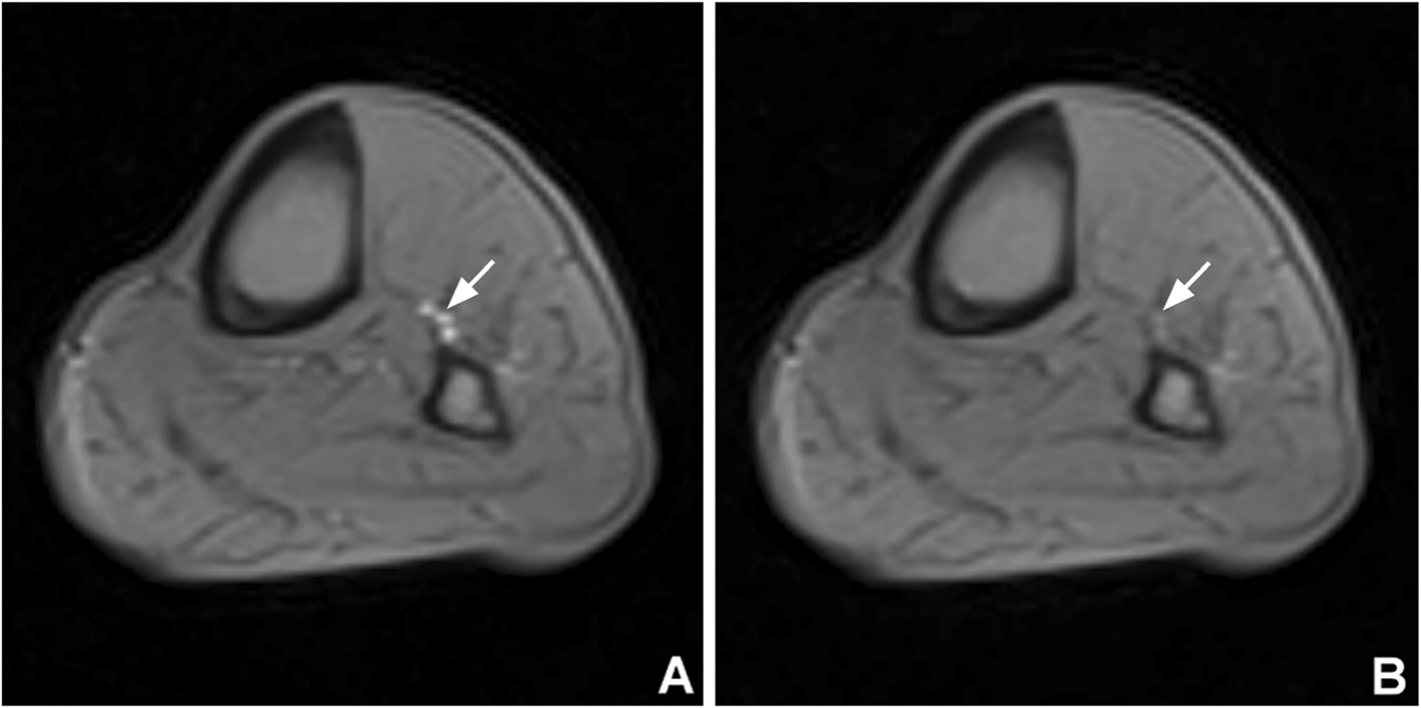
**SSFP cine images were obtained pre- (A) and post-cuff inflation (B) to check for effective arterial occlusion (arrows).** Pressure was increased up to 250 mmHg to achieve occlusion. SSFP = steady-state free precession.

Subjects performed supine plantar flexion exercise using a locally built MR-safe pedal ergometer [15] until exhaustion or had a thigh cuff inflated up to 250 mmHg for 5 min. Adequate arterial occlusion by the cuff was verified with an axial steady-state free precession (SSFP) cine image set at the level of the calf (Figure 1). PAD patients had their most symptomatic leg studied. Images were obtained using a flexible calf coil in a 3 T Trio scanner (Siemens Healthcare, Erlangen, Germany) immediately after cuff deflation or at end-exercise. Seven control-tagged image pairs were acquired over 60 seconds using a PASL pulse sequence with single-shot echo-planar imaging (EPI) readouts (field of view 200x200 mm, matrix 64x64, repetition time 4000 ms, echo time 32 ms, slice thickness 10 mm). PASL was performed using proximal inversion with control for off-resonance effects (PICORE) [16], which labels blood proximal to the imaged slice. The QUIPSS II with thin-slice TI_1_ periodic saturation (Q2TIPS) technique was used as previously described [15] in order to minimize systematic errors due to variable transit delay of spins and perfusion signal contamination by intravascular blood flowing through the slice. Signal averaging was performed with motion correction and relative blood flow maps representing perfusion over the imaging time were calculated online using a single compartment ASL model [17].

### Data analysis

**Figure 2:**
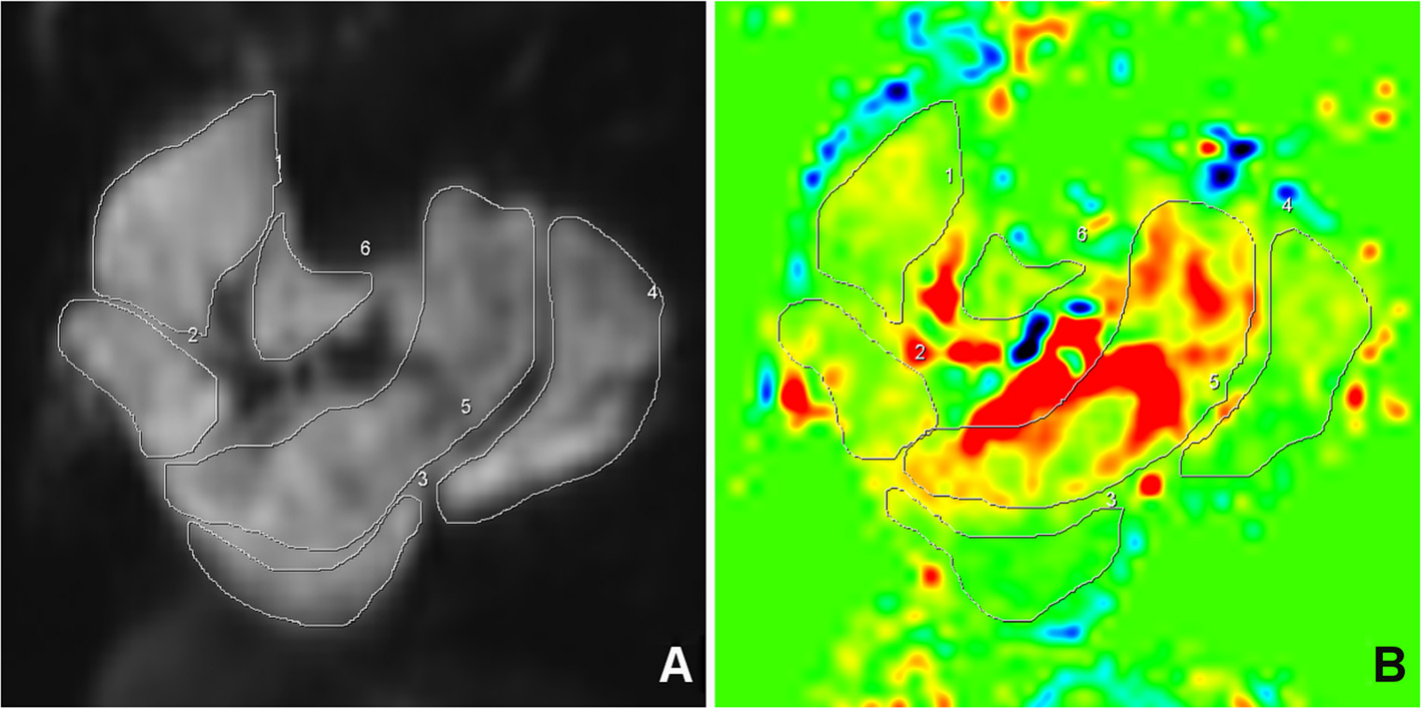
**Perfusion was measured by drawing regions of interest on the echo-planar images (A), which are then transferred to the perfusion maps (B) to obtain perfusion in ml/min-100 g.**

Perfusion was measured on a Leonardo workstation (Siemens Healthcare, Erlangen, Germany). Previously we measured perfusion by selectively drawing regions of interest (ROI) of the hyperemic areas on the perfusion maps [15]. To more readily compare exercise and cuff data in the present study, a different approach was taken. ROIs of each muscle group were initially drawn on the motion corrected EPI images while avoiding large vessels. The ROIs were then copied to the relative blood flow maps to obtain perfusion measurements in ml/min-100 g [17]. Perfusion map ROIs were then manually edited to avoid registration errors and imaging artifacts at the edge of the calf area (Figure 2). Cuff_*max*_ was defined as the muscle with the highest perfusion value. Cuff_*mean*_ was calculated by averaging all calf muscle group’s perfusion values. The Exercise data analysis was repeated using the same analysis method. Ten Cuff datasets were independently analyzed by two readers (DL, AJP) for measurement of interobserver agreement. Perfusion time curves with an effective temporal resolution of 8 s were derived from 10 Cuff (5 NL, 5 PAD) datasets using MATLAB 2012b (The MathWorks, Inc., Natick, MA). The average calf time to peak (TTP) perfusion was calculated.

### Statistical analysis

The primary outcome measure was calf perfusion at peak exercise and during post-occlusion hyperemia. The maximal exercise hyperemia was compared to both the maximal (Cuff_*max*_) and mean calf muscle post-occlusion hyperemia (Cuff_*mean*_). All patients’ baseline characteristics are presented as mean ± SD for continuous variables and n (%) for categorical variables. Kruskal-Wallis and Wilcoxon rank sum nonparametric tests were used as appropriate. Cuff interobserver and test-retest intraclass correlation coefficient (ICC) were calculated. Receiver-operator-characteristics (ROC) curves were generated and the area under the curve calculated for Exercise, Cuff_*max*_ and Cuff_*mean*_. All statistical analyses were performed using IBM SPSS Statistics for Windows, Version 21.0, Armonk, NY: IBM Corp.

## Results

### Patients

Baseline patient characteristics for the study are presented in Table 1. Fifteen NL subjects (54 ± 9 years) and 15 PAD (54 ± 7 years) patients exercised, while 12 NL subjects (55 ± 67 years) and 11 PAD patients (72 ± 11 years) underwent Cuff. PAD patients were somewhat older than healthy volunteers (67 ± 9 vs. 54 ± 8 years, p < 0.001). Five NL-Ex subjects and all NL-Cuff subjects had ABI testing (1.08 ± 0.06 and 1.00 ± 0.07 respectively). There was no difference in ABI between the PAD-Ex and PAD-Cuff groups (0.70 ± 0.14 vs. 0.68 ± 0.06; p = 0.286).

**Table 1:** **Patient characteristics**

**Characteristics**	**NL-Ex**	**NL-Cuff**	**PAD-Ex**	**PAD-Cuff**
	**(n = 14)**	**(n = 12)**	**(n = 15)**	**(n = 11)**
Age, yrs	54 ± 9	54 ± 6	64 ± 9	72 ± 11
ABI	1.08 ± 0.06	1.0 ± 0.07	0.70 ± 0.14	0.68 ± 0.05
Male	7 (50)	6 (50)	5 (33)	8 (73)
Active Tobacco use	0	0	8 (53)	5 (45)
Former tobacco use	0	0	3 (20)	5 (45)
Hypertension	0	0	12 (80)	10 (91)
Diabetes	0	0	7 (47)	6 (55)
Prior lower extremity stent	0	0	5 (33)	5 (45)
Prior lower extremity bypass	0	0	3 (20)	0

Mean ± SD or n (%). NL = healthy controls; PAD = peripheral arterial disease patients; Ex = exercise stress; Cuff = occlusion-hyperemia stress.

### Quantitative calf muscle perfusion

**Figure 3:**
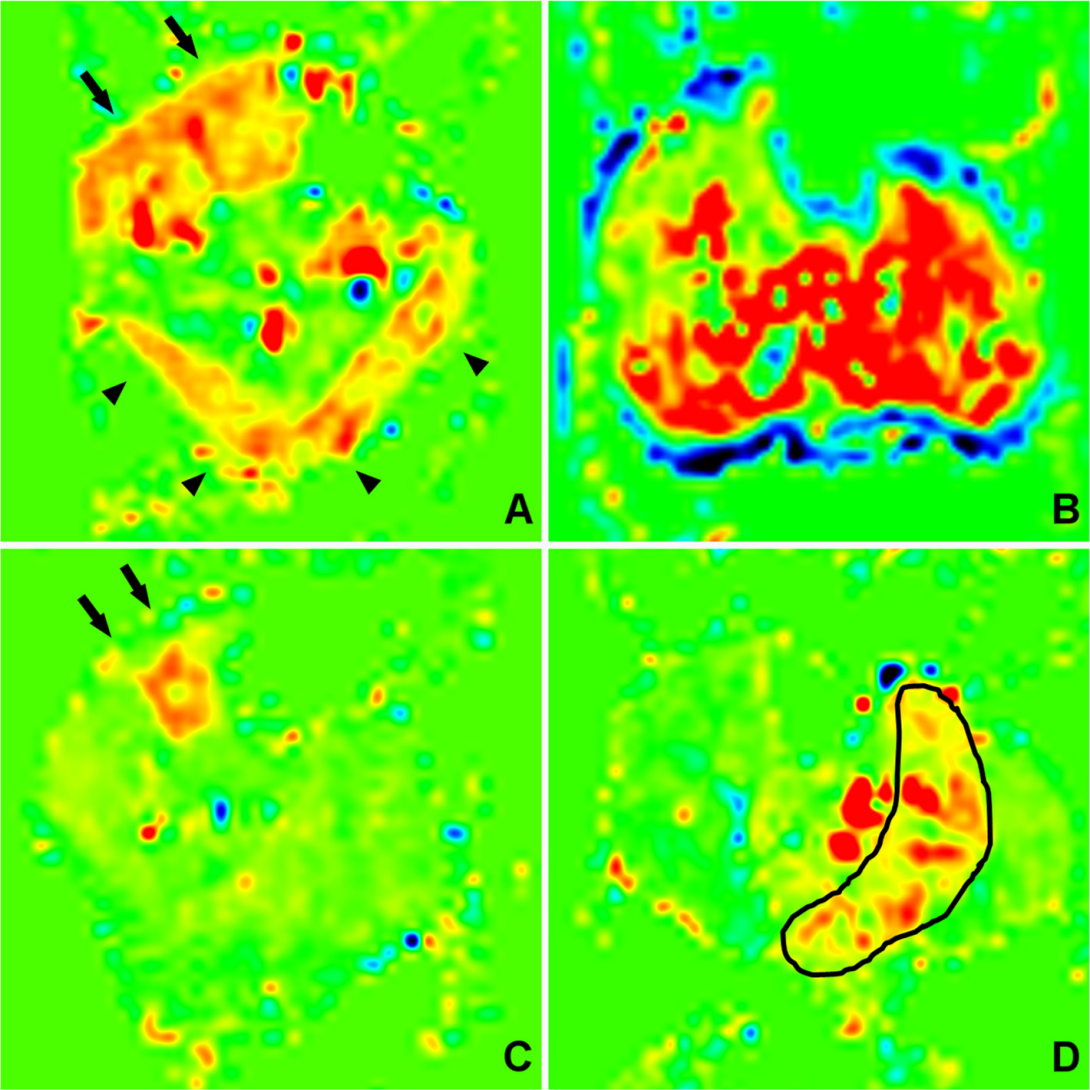
**Representative perfusion maps of each study group demonstrate the difference in perfusion patterns with exercise (A, C) compared to cuff occlusion hyperemia (B, D).** During exercise perfusion is limited to the anterior compartment, lateral compartment (arrows) and gastrocnemius (arrow heads). Post-occlusion hyperemia tends to be more diffuse in healthy volunteers **(B)**. However, in PAD patients **(D)** reactive hyperemia is of lesser magnitude and extent, generally limited to the soleus muscle (outlined).

**Figure 4:**
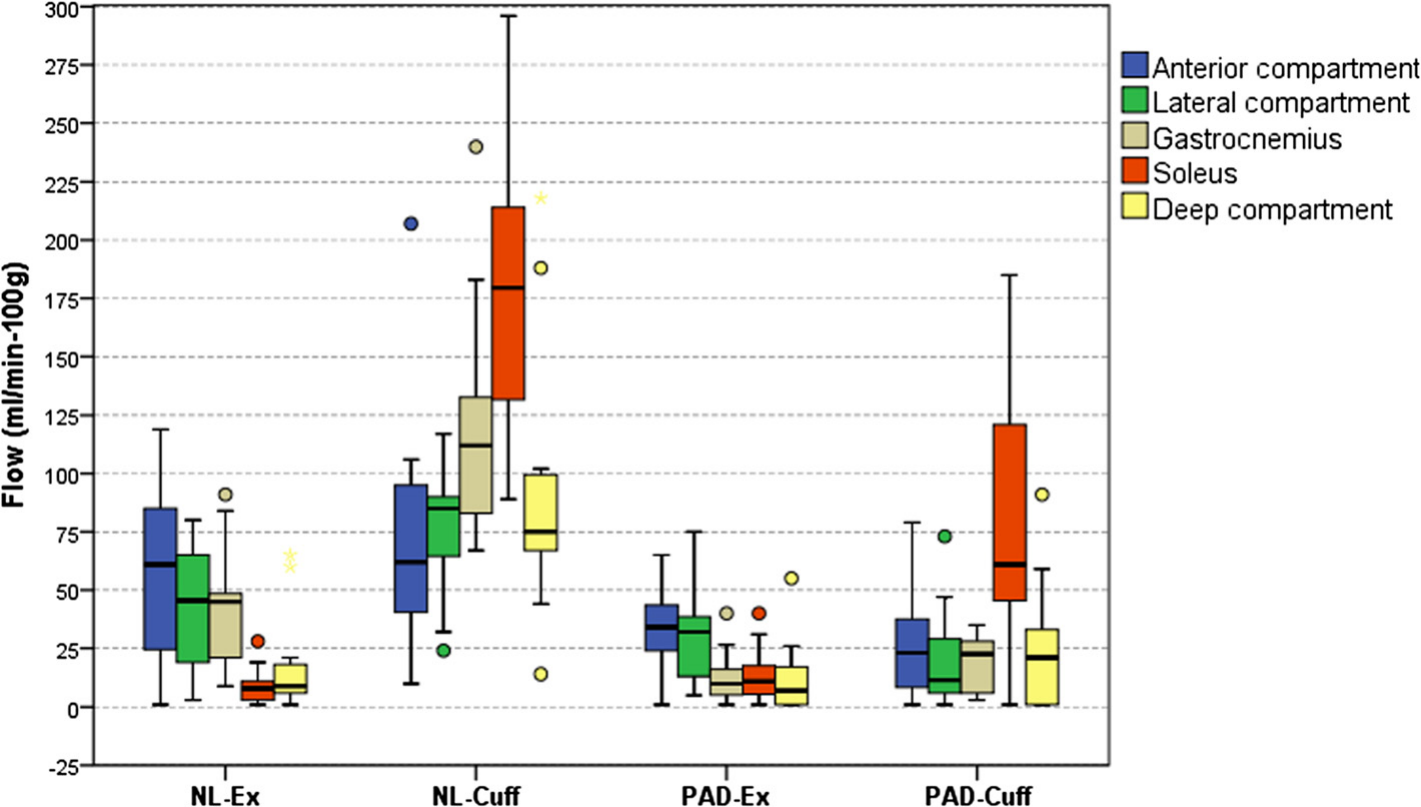
**The boxplots summarize muscle group perfusion results for all the study groups.** Circles indicate outliers. NL = controls; Ex = exercise hyperemia; Cuff = post-occlusion hyperemia; PAD = Peripheral arterial disease.

Representative perfusion maps of each study group are illustrated in Figure 3. At peak exercise, maximal hyperemia typically occurred in the anterior (AC) (46%) and lateral compartments (LC) (33%), followed by the gastrocnemius (17%) without significant increases in perfusion in the soleus and deep compartment (DC). Conversely, maximal post-occlusion hyperemia typically occurred in the soleus muscle (82%). While Cuff controls had substantial perfusion increases in all calf muscles, reactive hyperemia in PAD was generally limited to the soleus (Figure 4).

**Figure 5:**
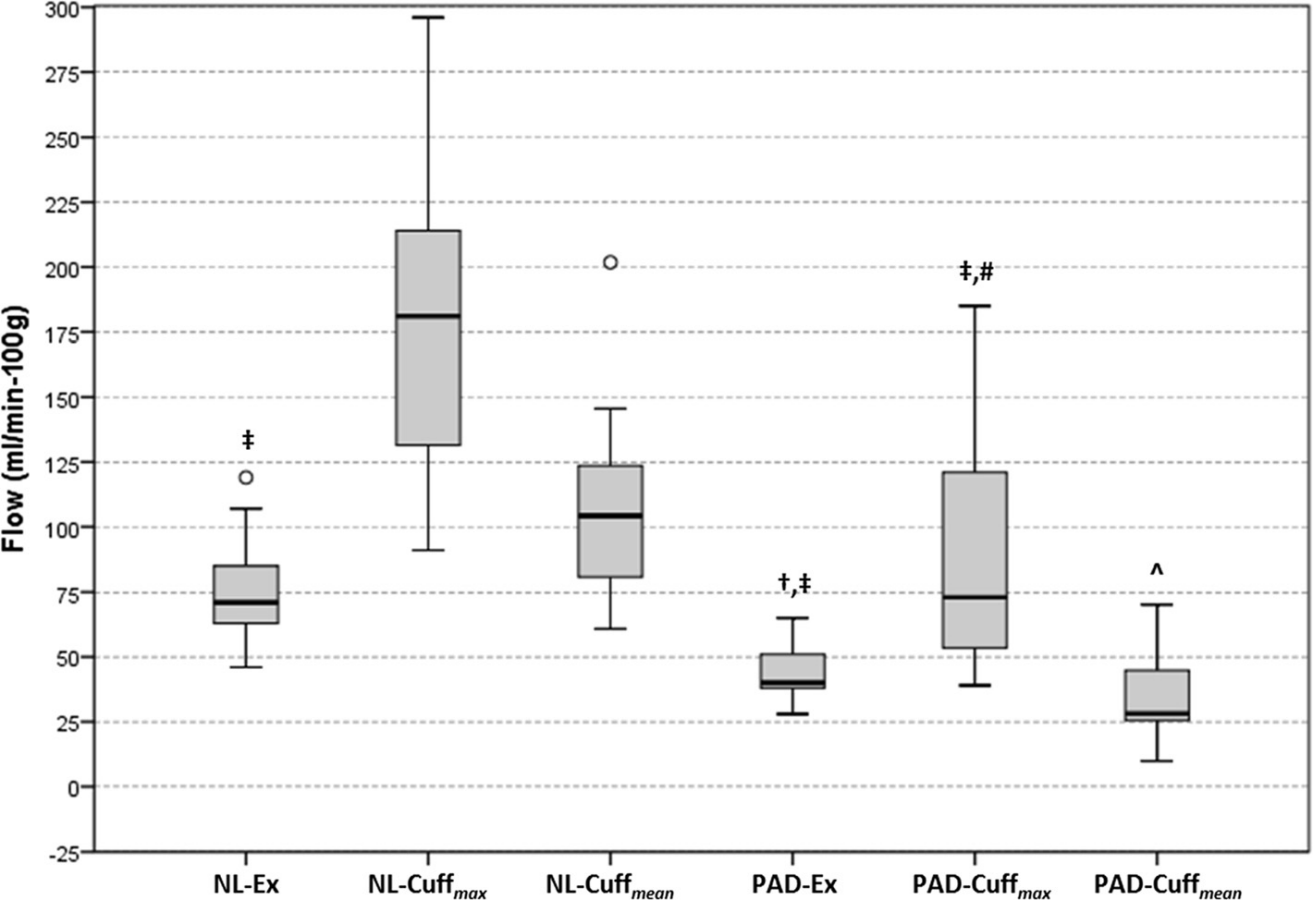
**Boxplot of maximal and average perfusion.** Circles indicate outliers. NL = controls; Ex = exercise hyperemia; Cuff_*max*_ = maximal post-occlusion hyperemia; PAD = peripheral arterial disease; Cuff_*mean*_ = average post-occlusion calf perfusion. ‡p < 0.05 vs. Max NL-Cuff; †p < 0.05 vs. Max NL-Ex; #p < 0.05 vs. Max PAD-Ex; ^p < 0.05 vs. Comp NL-Cuff.

The quantitative calf muscle perfusion results are summarized in Table 2. Both control groups had greater perfusion than PAD independent of stressor (NL-Ex 74 ± 21 vs. PAD-Ex 43 ± 10, p < 0.01; NL-Cuff_*max*_ 180 ± 60 vs. PAD-Cuff_*max*_ 90 ± 50 ml/min-100 g, p = 0.035; NL-Cuff_*mean*_ 109 ± 39 vs. PAD-Cuff_*mean*_ 34 ± 17 ml/min-100 g, p < 0.001)(Figure 5). Cuff occlusion elicited greater maximal hyperemia in both groups of subjects (NL-Cuff_*max*_ vs. NL-Ex, p = 0.012; PAD-Cuff_*max*_ vs. PAD-Ex; p < 0.001) driven by higher perfusion values in the soleus muscle. However, no within group differences were noted when comparing exercise to average cuff values (NL-Cuff_*mean*_ vs. NL-Ex, p = 0.611; PAD-Cuff_*mean*_ vs. PAD-Ex, p = 1.00). Notably, PAD-Cuff maximal (soleus) perfusion was no different than NL-Ex (PAD-Cuff_*max*_ 81 ± 59 vs. NL-Ex 74 ± 21 ml/min-100 g, p = 0.878). These results were mirrored in those subjects studied with both provocative tests (Exercise 83 ± 13, Cuff_*max*_ 142 ± 54, Cuff_*mean*_ 79 ± 36 ml/min-100 g).

**Table 2:** **Perfusion measurements by muscle group**

**Perfusion (ml/min-100 g)**	**NL-Ex**	**NL-Cuff**	**PAD-Ex**	**PAD-Cuff**
	**(n = 14)**	**(n = 12)**	**(n = 15)**	**(n = 11)**
Anterior compartment	52 ± 39	61 ± 33	33 ± 20	26 ± 24
Lateral compartment	44 ± 24	75 ± 27	26 ± 13	16 ± 15
Lateral gastrocnemius	38 ± 35	75 ± 14	6 ± 8	21 ± 16
Medial gastrocnemius	48 ± 24	127 ± 50	15 ± 11	22 ± 17
Soleus	9 ± 8	179 ± 60	13 ± 11	81 ± 59
Deep compartment	18 ± 21	95 ± 60	11 ± 15	28 ± 30
Maximal perfusion	74 ± 21	179 ± 60^†^	43 ± 10^†,‡^	92 ± 55^†,‡^
Average perfusion	-	109 ± 40	-	36 ± 19^‡^

^†^p < 0.05 vs. NL-Ex; ^‡^p < 0.05 vs. NL-Cuff. NL = healthy controls; PAD = peripheral arterial disease patients; Ex = exercise stress; Cuff = occlusion-hyperemia stress.

### Reproducibility and ROC

As previously published, exercise test-retest and interobserver ICC were 0.87 (95% confidence interval [CI]: 0.61 – 0.96) and 0.96 (95% CI: 0.84 – 0.99) respectively [15]. For the 8 Cuff subjects who returned for repeat testing, ICC was 0.98 (95% CI: 0.71-0.99). Cuff interobserver agreement was excellent with ICC of 0.99 (95% CI 0.98-0.99). The ROC areas for differentiating PAD and normal were 0.905 ± 0.061, 0.879 ± 0.070 and 0.992 ± 0.012 for Exercise, Cuff_*max*_ and Cuff_*mean*_ respectively.

### Cuff perfusion time curves

The average time to peak (TTP) perfusion was 14 s for NL and 23 s for PAD, indicating that the peak hyperemic perfusion was readily captured during the 60 second imaging period.

## Discussion

ASL CMR enables the measurement of calf muscle perfusion in healthy volunteers and PAD patients without the use of exogenous contrast in combination with exercise or cuff occlusion stress paradigms. We found that calf muscle perfusion is significantly higher in controls compared to PAD patients independent of stress modality. Perfusion was similar to exercise levels when Cuff perfusion was averaged. However, Cuff yielded greater maximal calf muscle perfusion than exercise within subject groups; an effect driven by higher perfusion values in the soleus. Soleus perfusion in the PAD-Cuff group was similar to exercise perfusion in normal subjects. When the same subjects underwent both stressors, perfusion was similar. Interobserver agreement was excellent for both methods, while test-retest reproducibility and area under the ROC curve were superior with Cuff.

Cuff occlusion has been reported to induce substantial increases in perfusion to all calf muscle groups [14,18,19]. However, there is significant heterogeneity in the magnitude of hyperemia with higher perfusion observed in the soleus muscle. In this study we show similar findings, with maximal Cuff perfusion occurring in the soleus in 83% of subjects. Using CE- and ASL-CMR we previously showed that supine ankle flexion-dorsiflexion exercise induces localized hyperemia in the anterior and lateral compartments and gastrocnemius muscles [7,15]. Consequently, we compared exercise hyperemia to maximal and average Cuff perfusion. Both parameters differentiated controls and PAD. Maximal perfusion values were greater with Cuff, driven by significantly larger hyperemic responses in the soleus muscle. Using average calf perfusion, perfusion within subject groups was similar. Furthermore, Cuff_*mean*_ showed higher reproducibility and better discriminatory properties.

Our findings confirm those of Wu et al. [14] indicating that in PAD patients reactive hyperemia is relatively preserved in the soleus and, although lower than NL-Cuff soleus, is similar to maximal NL-Ex perfusion. The mechanisms underlying this phenomenon are incompletely understood. The soleus receives dual blood supply from the posterior tibial and peroneal vessels. Additionally, it is a predominantly red muscle composed of Type I oxidative fibers. Animal studies have shown that oxidative muscles have increased capillary density and increased capillary to muscle fiber ratio compared to Type II glycolytic muscles such as the anterior tibialis [20]. Histopathological studies evaluating the soleus capillary anatomy in PAD patients is limited, but Askew et al. [21] have shown decreased Type I fibers and capillary density in the gastrocnemius muscle of patients with claudication. Thus, a combination of baseline differences between the soleus and other calf muscle capillary density, accentuated by adaptive responses in PAD, may be responsible in part for this finding.

The perfusion time course analysis indicates that the time to peak perfusion occurs relatively soon after cuff release for both normal subjects and PAD patients, albeit somewhat longer in PAD patients. This is consistent with prior studies using strain gauge plethysmography. Despite similar temporal resolution, these findings do not agree with those of Wu et al. who reported mean TTP of 45 – 55 s in NL, 65 – 76 s in moderate PAD and up to 86 – 99 s in those with ABI < 0.5 [14,18] when measured with CASL. Similarly, Grozinger et al. reported TTP of around 60s in claudicants with moderate to severely reduced ABI using pseudo-continuous ASL (PCASL) [22]. The reasons for the discrepancy in TTP results are unclear, but may be due in part to differences in experimental design and/or type of ASL used. We did not image throughout the occlusion period which may have caused a short delay in what we determined to be time zero.

There are multiple versions of ASL that fall into three primary types of preparation techniques to magnetically label inflowing blood protons: pulsed (PASL), continuous or pseudo-continuous (CASL, PCASL), and velocity-selective ASL [23]. In our study we used PASL utilizing the PICORE and Q2TIPS techniques. Advantages of PASL over CASL include lower radiofrequency (RF) power deposition, and the absence of need for continuous RF transmit hardware [17,24], which is not readily available in most centers. PCASL does provide higher signal-to-noise ratio and does not require specialized hardware. Hence, it has been recommended as the preferred mode for brain perfusion imaging [23]. Its application to calf perfusion appears promising as demonstrated in a study calf perfusion changes before and after percutaneous angioplasty in a pilot study of 10 claudicants [22].

The ability to quantitatively measure calf perfusion without the use of gadolinium is an important advantage over CMR because of the high prevalence of advanced CKD [25] in PAD patients who are most vulnerable to nephrogenic systemic fibrosis [9]. Another benefit of a contrast-free technique is the elimination of the need for blood chemistry tests and intravenous line placement. Lastly, with this particular sequence, perfusion maps are instantly reconstructed online, and easily analyzed at the MR console.

### Optimal stress modality

Because skeletal muscle perfusion at rest is extremely low in both controls and PAD patients, ASL cannot be used to quantify rest perfusion as perfusion values are at the level of noise in the images. Hence, ASL requires a physiological challenge to measure skeletal muscle tissue perfusion. Exercise and cuff occlusion are commonly used methods, but it has been unclear if one is superior to the other. Our results would indicate that cuff occlusion improves upon the test-retest reproducibility of exercise. The superior reproducibility of Cuff may be mediated by the elimination of the effort-dependent component of exercise stress. Interobserver agreement was excellent with both provocative tests.

Although exercise stress has somewhat lower reproducibility, it has close clinical correlates. Perfusion CMR at peak exercise has been shown to correlate with walking distance [8] and time to claudication [26]. Studies correlating functional capacity and Cuff have not been performed. However, PAD patients with CLI or arthritis, may be unable to adequately exercise. Furthermore, cuff occlusion elicits hyperemia beyond the muscles used during supine ankle exercises, which would allow studies of regional perfusion throughout the calf.

The optimal stress modality may ultimately be dependent on the patient cohort and the question at hand. Exercise may be useful for studies of therapies targeting improved perfusion in ambulatory patients with claudication; especially those utilizing exercise training. On the other hand, trials of therapies targeting CLI in patients who are generally unable to exercise may be best served by Cuff. In addition, a better discriminatory power and reproducibility suggests that Cuff_*mean*_ may be the parameter of choice to study populations at risk such as young smokers and diabetics with borderline ABI.

### Limitations

Exercise and cuff occlusion hyperemia are physiologically different entities and interpretation of the results is dependent on the analytic approach. The exercise used for this study does not elicit hyperemia in the soleus; therefore it is not known how exercise-induced soleus perfusion compares to cuff occlusion. Secondly, the primary outcome measures were obtained comparing exercise to Cuff in two different patient cohorts of similar clinical classification. However, results were similar in a subgroup that underwent testing with both stressors. Third, our PAD cohort was made up of patients with mild to moderate disease and a study of the feasibility of Cuff in patients with CLI is needed. In addition, Cuff occlusion is not applicable in patients with superficial femoral artery stents. Finally, we did not perform angiography or segmental pressures to localize disease.

## Conclusions

PASL cuff occlusion can differentiate PAD patients from controls with excellent test-retest and interobserver reproducibility. Given its physiologic nature and established clinical correlations, exercise may be useful for PASL-CMR studies of specific PAD therapies in claudicants. However, due to its superior reproducibility, PASL cuff occlusion may be the preferred approach.

## Abbreviations

ASL: Arterial spin labeling
CASL: Continuous arterial spin labeling
CLI: Critical limb ischemia
CMR: Cardiovascular magnetic resonance
Cuff: Cuff occlusion
Cuff_*mean*_: Average calf cuff occlusion hyperemia
Cuff_*max*_: Maximal calf cuff occlusion hyperemia
ICC: Intraclass correlation coefficient
NL: Healthy volunteers
NL-Cuff: Healthy volunteers tested with cuff occlusion
NL-Ex: Healthy volunteers tested with exercise stress
PAD: Peripheral arterial disease
PAD-Cuff: PAD patients tested with cuff occlusion
PAD-Ex: PAD patients tested with exercise
PCASL: Pseudo-continuous arterial spin labeling
PASL: pulsed arterial spin labeling
ROC: receiver operator characteristics
T: Tesla
